# Computational Study of ZnO Surface Catalysis: Adsorption of H_2_O or/and O_2_ as a Pathway to ROS Formation

**DOI:** 10.3390/nano15171328

**Published:** 2025-08-29

**Authors:** Sena E. Adjovi, Monica Calatayud, Lourdes Gracia

**Affiliations:** 1 Sorbonne Université, CNRS, MONARIS, CNRS-UMR 8233, 4 Place Jussieu, F-75005 Paris, France; sena.adjovi@etu.sorbonne-universite.fr (S.E.A.); monica.calatayud@sorbonne-universite.fr (M.C.); 2 Departament de Química Física, Universitat de València, 46010 Burjassot, Spain

**Keywords:** ZnO surfaces, adsorption, ROS, DFT

## Abstract

Reactive oxygen species (ROS) play a central role in photocatalytic processes relevant to environmental remediation and clean energy. This work focused on the computational investigation of ZnO surface reactivity toward H_2_O and O_2_ adsorption, as a preliminary step in understanding ROS generation pathways. Surface stability and adsorption energies for isolated and co-adsorbed H_2_O and O_2_ molecules on different ZnO surfaces (both in their pristine form and with oxygen vacancies) were evaluated using DFT calculations at the PBE-D3 level under various surface coverages. The introduction of vacancies on the pristine (001) and (100) surfaces enhanced O_2_ binding, particularly in inclined configurations at the defect sites, with the adsorption energies reaching −2.63 eV and −2.04 eV, respectively. However, the (110) surface showed very strong H_2_O binding, but weak O_2_ adsorption, which only modestly improved with vacancies. Co-adsorption of H_2_O and O_2_ exhibited synergistic stabilization, especially on the (001) and (100) surfaces, where ROS were formed through proton transfers either between adsorbed H_2_O and O_2_ or between H_2_O and surface oxygen atoms. These findings provide detailed insight into the mechanistic role of surface defects in ROS generation and support the rational design of ZnO-based photocatalysts.

## 1. Introduction

Photocatalysis has emerged as a sustainable approach to address both environmental pollution and the growing demand for clean energy. By harnessing light to activate chemical reactions [1], it enables the degradation of harmful contaminants in air and water, as well as the production of clean fuels, such as hydrogen from water [2,3]. Water pollution remains a critical global issue due to increasing contamination from industrial, agricultural, and pharmaceutical sources. While conventional treatment methods such as filtration, chlorination, and ozonation are widely used, they often prove insufficient against persistent pollutants and may generate toxic by-products [3]. To address these challenges, heterogeneous photocatalytic treatment has gained increasing attention since it works under mild conditions, requires no chemical additives, and relies on renewable energy, making it an eco-friendly and sustainable solution [4]. Central to this process are semiconductor materials, whose unique electronic properties enable the absorption of light and the subsequent chemical reactions that drive photocatalysis. In fact, new paths in the development of sustainable technologies for recycling the Earth’s resources and contributing to a circular economy using semiconductor materials have been reported [5,6,7,8].

When a semiconductor is irradiated with light whose energy equals or exceeds its band gap, an electron (*e^−^*) gains enough energy to jump from the valence band to the conduction band, leaving behind a positively charged hole (*h^+^*). This *e^−^/h^+^* pair is the engine of photocatalysis. Once generated, the electron and hole can migrate to the surface of the material and participate in redox reactions: the electron can reduce species like protons (H^+^) into hydrogen gas (H_2_), while the hole can oxidize water molecules or organic pollutants [3,9,10]. Metal oxides have emerged as the most widely used semiconductor photocatalysts, especially for environmental applications [1,11], since their strong oxidative power, particularly under ultraviolet (UV) light, makes them well-suited for breaking down stubborn organic pollutants [12]. Among metal oxides, ZnO stands out as one of the most promising materials for photocatalysis. ZnO possesses a wide band gap of approximately 3.37 eV at room temperature [13], making it effective at absorbing UV light and generating electron–hole pairs. In addition, it has a high exciton binding energy (~60 meV) [14], which contributes to the efficient generation and separation of charge carriers and is a critical factor in ensuring that electrons and holes can participate in redox reactions before recombining. ZnO is also versatile in terms of its morphology: it can be synthesized in various nanostructured forms, such as nanorods, nanowires, or nanoparticles, which increases the surface area and enhances its photocatalytic efficiency [15]. Moreover, ZnO is low-cost, non-toxic, and biocompatible, making it attractive for applications ranging from wastewater treatment to biomedical uses.

In light of these advantageous properties, the effectiveness of materials like ZnO in heterogeneous photocatalysis is ultimately linked to the formation of reactive oxygen species (ROS). These species play a central role in driving the chemical reactions initiated upon light absorption. Although often associated with oxidative stress in biological systems, in the field of photocatalysis, ROS are indispensable intermediates that drive many of the key transformations [16]. These species are responsible for initiating and sustaining the oxidative or reductive reactions that underpin both pollutant degradation and energy conversion processes. Understanding their nature and how they form provides essential insight into the effectiveness of photocatalytic systems. The generation of ROS in a photocatalytic system begins with the absorption of photons by the semiconductor, creating *e^−^/h^+^* pairs. These charge carriers then migrate to the surface of the photocatalyst, where they interact with adsorbed species such as oxygen and water. Typically, the photogenerated holes can oxidize water molecules or hydroxide ions to produce hydroxyl radicals (•OH), whereas the photogenerated electrons reduce molecular oxygen to superoxide radicals (•O_2_^−^) [3]. Several types of ROS can be generated during photocatalysis, each with distinct reactivity [16,17]. These species vary in terms of their oxidative strength and lifetime, but all play significant roles depending on the photocatalyst used and the surrounding environment. For example, hydroxyl radicals are known for their high reactivity and ability to non-selectively oxidize a wide range of organic compounds, while superoxide anions tend to participate in stepwise reduction processes [18]. Depending on the reaction pathways and environmental conditions (e.g., pH and dissolved oxygen content), further reactions can lead to the formation of other ROS. •O_2_^−^ and •OH species can transform into other highly oxidative species: singlet oxygen (^1^O_2_), perhydroxyl radical (•O_2_H), or hydrogen peroxide (H_2_O_2_) [19]. In water splitting for hydrogen production, for instance, ROS can facilitate intermediate steps or act as indicators of surface reactivity. In addition, recent studies reported that ultrafast hydrogen detection systems are based on sensor response processes [20,21]. In environmental remediation, they are directly responsible for the breakdown of persistent organic pollutants and microbial contaminants, or helping to inhibit bacteria growth [22].

The present work aims to provide a computational insight into the catalytic activity of ZnO surfaces as potential platforms for the generation of ROS, through the adsorption and interaction of H_2_O and O_2_ molecules. Using Density Functional Theory (DFT), this study seeks to model and predict, at the atomic scale, the initial stages that govern ROS production on ZnO surfaces, a process central to both environmental remediation and biomedical applications. To accomplish this, several key aspects are investigated. Firstly, the surface stability of various crystallographic facets of ZnO is assessed in order to determine the most energetically favorable planes for catalytic processes. Based on these findings, the equilibrium morphology of a ZnO nanocrystal is constructed to better reflect realistic surface environments. Secondly, the focus is placed on the interaction of H_2_O, O_2_, and their co-adsorption (H_2_O + O_2_) on the most stable surfaces. For each case, optimized adsorption geometries and adsorption energies are calculated, along with an analysis of the charge transfer phenomena between the surface and the adsorbed species. This allows for the identification of favorable electronic interactions that may initiate ROS formation. Additionally, oxygen vacancies are introduced into the most stable surfaces to evaluate their influence on adsorption behavior and electronic structure, as well as their role in the catalytic reactivity of ZnO surfaces. Finally, the thermodynamics of H_2_O dissociation into hydroxyl and proton species on the surface is explored. Special attention is given to the transfer of protons from adsorbed water molecules either to surface oxygen atoms or to the oxygen atoms within adsorbed O_2_, in order to evaluate the energetic viability and stability of likely ROS intermediates.

## 2. Computational Methods

The ZnO structure was optimized using the Vienna ab initio Simulation Package (VASP) [23] within the DFT framework. The generalized gradient approximation (GGA), in combination with the exchange correlation functional by Perdew, Burke, and Ernzerhof (PBE) [24] with Grimme’s DFT-D3 [25] dispersion correction, was used for all bulk and surface optimizations to assess the structural and energetic accuracy. A 4 × 4 × 4 Monkhorst-Pack k-point grid was applied, and the core electrons of ZnO were treated using pseudopotentials built from the Projector Augmented Wave (PAW) method [26], such that the valence configuration for Zn was 4d^10^5s^2^, and for O, it was 2s^2^2p^4^. In systems containing hydrogen, the 1s^1^ configuration was considered. Among the three main polymorphs of ZnO (wurtzite [27], zinc blende [28], and rocksalt [28]), the wurtzite structure is the most prevalent in both natural and synthetic forms, and it is especially suited for photocatalytic applications thanks to **its** intrinsically polar surfaces and favorable electronic properties [29]. The initial ZnO crystal structure corresponding to the wurtzite phase (space group P6_3_mc) was obtained from ICSD [27], with experimental lattice parameters a = b = 3.24 Å and c = 5.22 Å [30].

Surface slab models were constructed for seven crystallographic planes—(001), (100), (2–10), (101), (102), (111), and (112)—based on relevant low-index and high-index facets. A vacuum of 15 Å was applied along the z-axis, and slab thicknesses ranged from 10 to 20 Å to ensure surface energy convergence (see Figure S1 of Supplementary Material). Surface energies (γ) were calculated using a standard expression:

(1) $$\gamma =\frac{1}{2A}\;\left(E_{slab}-n\cdot E_{bulk}\right)$$

where *A* is the area of the surface unit cell, *E*_slab_ is the energy of the slab supercell, *E_bulk_* is the bulk energy, and *n* is the number of bulk units in the slab. The factor of ½ accounts for the presence of two surfaces in each slab. The most stable slab was selected for each facet. The recommended cut-off of 400 eV for the plane wave basis sets was applied to the valence electrons, as well as a Gaussian smearing of 0.05 eV. The tetrahedron method with Blöchl corrections was used to obtain the final static simulations to ensure accurate total energies, and the criterion for the electronic optimization was set at 10^−5^ eV. Morphological predictions were obtained via Wulff constructions performed in the VESTA3 software.

To investigate adsorption phenomena, supercells of increasing size were built for selected surfaces to simulate various molecular coverages of H_2_O and O_2_. Defect models included a single surface oxygen vacancy (V_O_) per supercell. Adsorption energies ${(E}_{ads})$ were computed as

(2) $$E_{ads}=E_{slab\_molec}-E_{slab}-E_{molec}$$

where $E_{slab\_molec}$ is the total energy of the optimized adsorption system (i.e., ZnO slab (pristine or with an oxygen vacancy) with the adsorbate species), $E_{slab}$ is the energy of the pristine/defective substrate (supercell), and $E_{molec}\;$ is the energy of the isolated adsorbate molecule (O_2_ or H_2_O).

Spin-polarized calculations were performed for systems involving O_2_, while non-spin-polarized settings were used for H_2_O. The geometries of adsorbed species were optimized from various initial orientations and distances. The energies of isolated H_2_O and O_2_ molecules were computed in large vacuum cells to ensure that there is no interaction with adsorbed molecules. Bader charge analysis was conducted in the most stable systems to quantify electron transfer.

Finally, co-adsorption scenarios were explored by placing O_2_ on pre-adsorbed H_2_O–ZnO surfaces, followed by full optimization. Co-adsorption energies were computed to assess stability and interaction effects. Two adsorption energy expressions were employed. The first,$\;E_{coads}^{1}$, was used to assess the overall stability of the co-adsorbed system relative to its separate components:

(3) $$E_{coads}^{1}=E_{slab\_H2O\_O2}-E_{slab}-E_{H2O}-E_{O2}$$

The second expression, $E_{coads}^{2}$, allowed for the evaluation of O_2_ stabilization in the presence of pre-adsorbed H_2_O—the relevant scenario for this study:

(4) $$E_{coads}^{2}=E_{slab\_H2O\_O2}-E_{ZnO\_H2O}-E_{O2}$$

where $E_{ZnOs\_H2O\_O2}$ is the total energy of the co-adsorbed system, with both H_2_O and O_2_ adsorbed on the ZnO surface, after full structural relaxation. $E_{ZnOs\_H2O}$, $E_{ZnOs}$,$\;E_{O2}$, and $E_{H2O}$ is, respectively, the total energy of the ZnO surface with one water molecule adsorbed, the energy of the pristine ZnO surface supercell, and the energy of an isolated O_2_ and H_2_O. The thermodynamics of adsorbed H_2_O dissociation into adsorbed OH+H was also explored to study the potential formation of reactive oxygen species (ROS), with full relaxation of the resulting configurations.

## 3. Results and Discussion

### 3.1. Structure Optimization and Morphology

**Figure 1:**
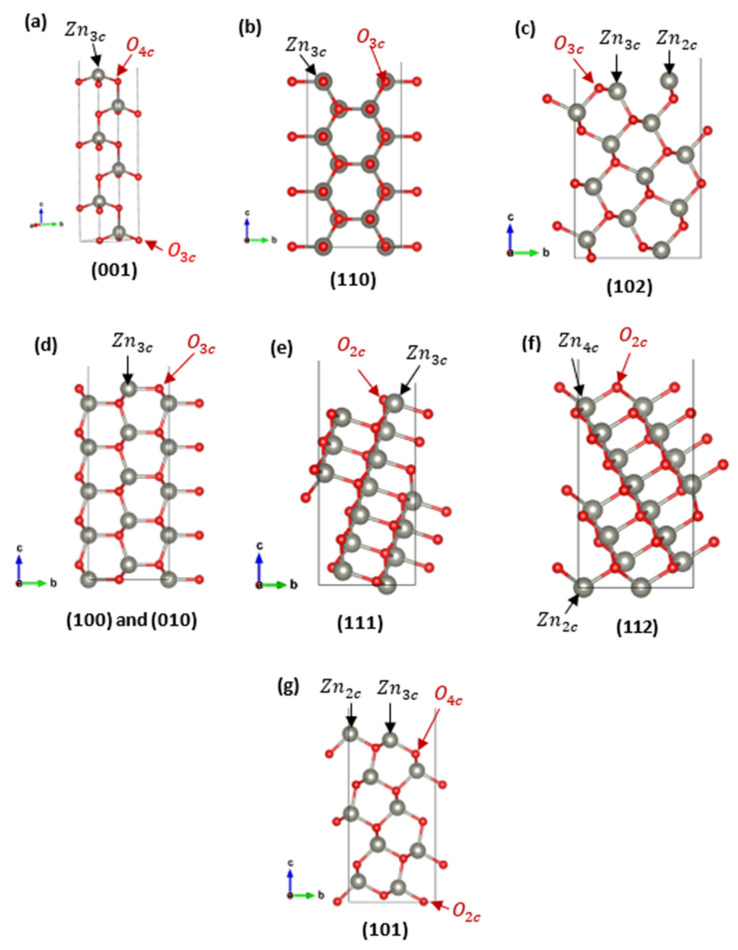
Calculated ZnO slab models showing the investigated surface terminations and the coordination numbers of surface atoms (Zn_1_–_4_c, O_2_–_4_c) for each crystallographic facet.

The optimized structural parameters of ZnO (a = 3.2470 Å and c = 5.2006 Å) are reflected in a consistent c/a ratio close to the ideal experimental value (~1.60) [26], with deviations Δa = 0.16% and Δc = 0.25% along the a and c axes, respectively. The different surface slab models investigated in this study are presented in Figure 1. All the constructed models are stoichiometric, but they differ in symmetry: while most surfaces are symmetric, a few, namely, (001), (112), and (101), are asymmetric, meaning that the two exposed terminations at either end of the slab are chemically distinct. In these asymmetric models, both surface terminations were analyzed individually, with surface images showing either a Zn-terminated or O-terminated configuration where applicable.

According to the surface energy values listed in Table 1, the stability of these surfaces can be ranked in ascending order of surface energy (i.e., from most to least stable) as follows: (010) ≈ (100) > (110) > (111) > (102) > (001) > (101) > (112).

This ordering provides essential insight into the relative thermodynamic stability of each facet. In particular, the low-index non-polar surfaces, (010), (100), and (110), exhibit the lowest surface energies, confirming their natural tendency to be expressed in the equilibrium morphology. On the contrary, surfaces such as (112) and (101) are significantly less stable, which reduces their probability of being present unless stabilized by external conditions such as adsorbates or growth kinetics.

**Figure 2:**
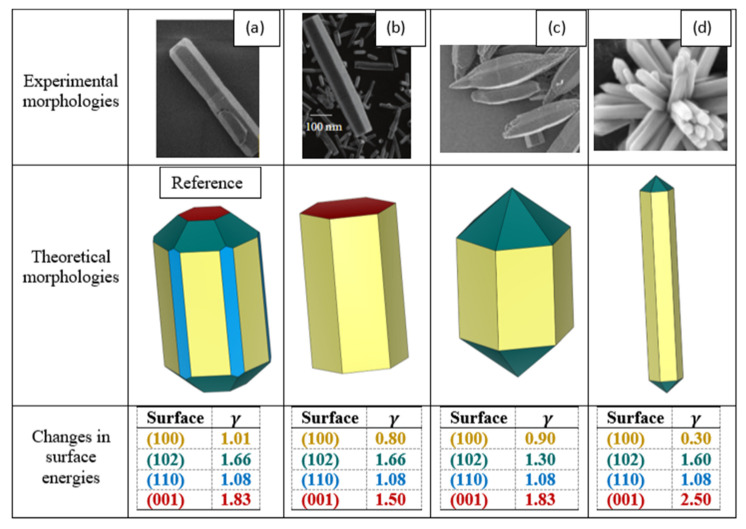
Reference morphology of ZnO derived from DFT-calculated surface energies $\gamma (J/m^{2})$, compared with three adjusted theoretical shapes obtained by variation in surface energies to reproduce experimentally observed nanostructures: (**a**) truncated [31], (**b**) rod-like [32], (**c**) tip-like [33] and (**d**) flower-like [34]. Reprinted with permission from [31,32], under the terms of the Creative Commons CC—BY license. Reprinted with permission from [33]; Copyright Clearance Center’s RightsLink^®^ 2020, Elsevier. Reprinted with permission [34]; Copyright Clearance Center’s RightsLink^®^ 2011 WILEY-VCH.

The computed surface energies serve as inputs for constructing the equilibrium crystal morphology of ZnO using the Wulff construction. According to Wulff’s theorem, each facet’s distance from the origin in the constructed morphology is proportional to its surface energy. The resulting morphology, illustrated in Figure 2 as a reference, reveals that only the four most exposed surfaces, (100), (001), (110), and (102), are expressed in the equilibrium shape. The equivalence of (010) and (100) arises from the hexagonal symmetry of the wurtzite structure (a = b), and these facets are therefore interchangeable in the morphology. This morphology (Reference), which we obtained based on surface energy calculations, has already been experimentally observed (Figure 2a) by Debroye et al. [31] under standard growth conditions, and is indeed one of the well-documented truncated shapes for ZnO nanocrystals. From this primary structure, three additional morphologies, also reported in experimental studies, can be reproduced by strategically modifying the surface energies of specific facets. A comprehensive comparison between experimental and theoretical morphologies of ZnO nanocrystals is performed, demonstrating a strong correlation between observed shapes and those predicted by our surface energy model.

The morphology illustrated in Figure 2b [32], characterized by a pronounced hexagonal rod-like elongation, results from the preferential stabilization of the (001) and (00-1) facets via an estimated ~18% reduction in their surface energies, while the (100) side facet is stabilized via a ~26% reduction. This suggests that the synthesis conditions, such as pH adjustment or the presence of specific dopants, as discussed by Amine et al. [32], led to the observed anisotropic growth by selectively stabilizing these three facets, thus governing the resulting morphology (b).

In contrast, morphology (c) [33] evolves into a more compact and tip shape as a result of moderate stabilization of the (102) and (10-2) facets via an estimated ~21% reduction in their surface energies, while the (100) side facet is stabilized via a ~17% reduction. This implies that changes in solvent polarity or precursor concentration, as reported by Wu et al. [28], lead to the stabilization of these three facets, thus governing the final crystal shape (c).

Finally, morphology (d) [34], characterized by its radial, flower-like appearance, is reproduced by drastically stabilizing the (100) facet (~70% decrease in surface energy) and significantly destabilizing the (001) and (00-1) facets (~37% increase)**.** These extreme modifications are consistent with surfactant-mediated synthesis or high ionic strength conditions as reported by Zhang et al. [35], which suppress polar (001) facet growth and promote lateral expansion, leading to this flower-like morphology (d).

Overall, this analysis underscores the predictive power of surface energy modeling in nanocrystal shape control and consequently strengthens our confidence in the selection of surface models for subsequent adsorption studies, ensuring that simulations are focused on the most relevant and realistic configurations.

### 3.2. Adsorption of H_2_O and O_2_ on ZnO Surfaces

To assess the surface reactivity of ZnO toward H_2_O and O_2_ molecules, a comprehensive set of adsorption simulations was carried out on the (100), (110), and (001) facets. These surfaces were chosen based on their prominence in the Wulff-constructed morphologies.

#### 3.2.1. Adsorption Characteristics of H_2_O and O_2_ on ZnO (001) Surface

**Figure 3:**
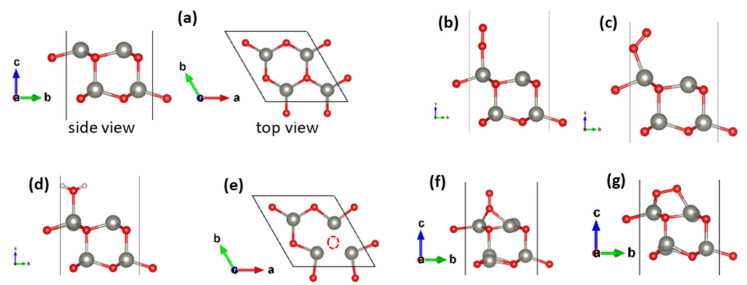
Optimized geometries of various adsorption configurations on ZnO (001) surfaces: (**a**) top/side view of pristine 2 × 2; (**b**–**d**) apical O_2_, inclined O_2_ and H_2_O adsorption; (**e**) top view of vacancy (dotted circle) 2 × 2 (001); (**f**,**g**) apical and inclined O_2_ adsorption at vacancy sites (identical geometry for all the other supercells).

To investigate how molecular orientation and surface coverage affect the adsorption behavior of O_2_ on the pristine ZnO (001) surface (Figure 3a), adsorption energies were computed for two configurations, apical and inclined at three different coverages (12%, 25%, and 50%) using 2 × 4, 2 × 2, and 1 × 2 supercells. The relaxed geometries reflect the optimization of initial placements atop Zn sites (Figure 3b,c).

Adsorption energies were computed at 12%, 25%, and 50% coverage using 2 *×* 4, 2 × 2, and 1 *×* 2 supercells (see Figure S2 of Supplementary Material). The adsorption energy results (Table 2) show a clear dependence on both coverage and molecular orientation. In all cases, the inclined (Figure 3c) configuration is energetically more favorable than the apical one (Figure 3b). For instance, at 12% coverage, the inclined adsorption energy reaches −1.11 eV, compared to only −0.59 eV for the apical case. As the surface becomes more saturated (from 12% to 50% coverage), the adsorption energy becomes less negative for both orientations, indicating a weakening of the O_2_–surface interaction due to increased intermolecular repulsion and reduced availability of optimal adsorption sites.

For H_2_O (Figure 3d), adsorption on the pristine surface yields moderate interaction strengths, with adsorption energies ranging from −0.81 eV to −0.87 eV across coverages. Unlike O_2_, the variation with coverage is weak, suggesting that H_2_O experiences less lateral interaction, likely due to hydrogen bond rather than intermolecular repulsion. Overall, the pristine (001) surface presents balanced affinity for both O_2_ and H_2_O, with a clear preference for the inclined O_2_ configuration.

**Figure 4:**
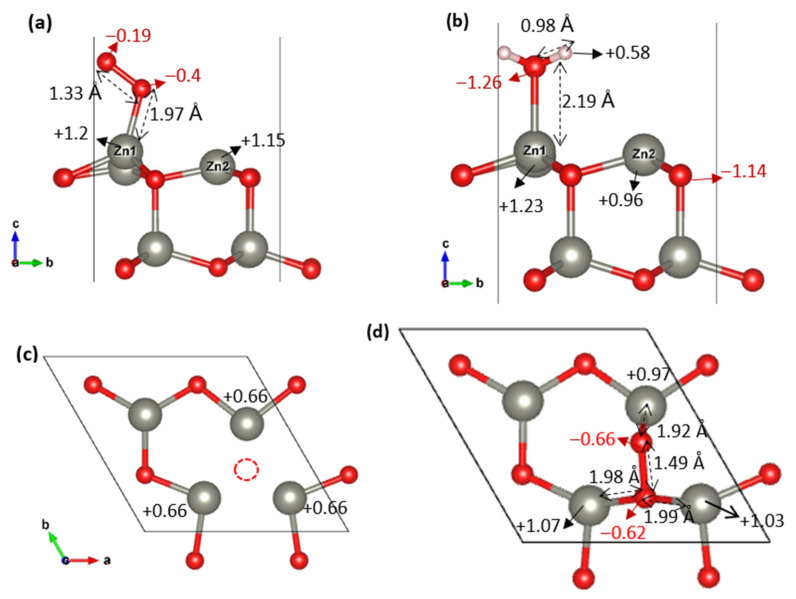
Optimized geometries and charge distributions (|e|) for H_2_O and O_2_ adsorption on the ZnO (001) 2 × 2 surface: (**a**) O_2_ (inclined) on the pristine surface, (**b**) H_2_O on the pristine surface, (**c**) top view of vacancy (dotted circle) and (**d**) O_2_ (inclined) on the vacancy fold.

To assess the effect of oxygen vacancies (Figure 3e), four distinct O_2_ configurations were tested on the ZnO (001) surface containing a surface O vacancy: (1) apical on a Zn site, (2) inclined on a Zn site, (3) apical on the vacancy site (Figure 3f), and (4) inclined on the vacancy site (Figure 3g).

The presence of the vacancy significantly enhances O_2_ adsorption compared to the pristine surface (Table 3). Among all configurations, the inclined adsorption on the vacancy site is the most favorable, reaching −2.63 eV at 12% coverage, far surpassing both the Zn-site adsorption (−0.90 eV inclined, −0.83 eV apical) and the apical adsorption on the vacancy (−1.50 eV). As surface coverage increases, the adsorption energy on the vacancy site becomes less negative (−2.33 eV at 25%, −1.98 eV at 50%), reflecting slightly reduced interaction strength due to site saturation and intermolecular repulsion, but it remains substantially stronger than adsorption on pristine Zn sites.

For H_2_O, adsorption on the vacancy surface yields moderate adsorption energies (−0.49 to −0.66 eV), which are weaker than on the pristine surface (−0.81 to −0.87 eV). This indicates that, unlike O_2_, the presence of oxygen vacancies actually destabilizes H_2_O adsorption, likely due to the loss of hydrogen bonding interactions between the water hydrogen and surface oxygen atoms, which are disrupted by the absence of lattice oxygen at the vacancy site.

The stability trend observed in adsorption energies between pristine and defective ZnO surfaces can be directly correlated with charge transfer mechanisms and local adsorption geometry. On the pristine surface, both O_2_ and H_2_O exhibit net charge gains upon adsorption, reflecting electron donation from the surface to the adsorbates. In the case of H_2_O (Figure 4b), the total charge gain is modest (−0.10 |e|), while the O_2_ molecule in the inclined configuration (Figure 4a) accumulates approximately −0.59 |e| (−0.40 and −0.19 |e| on each oxygen atom). This difference suggests that O_2_, although more electronegative, induces a stronger surface polarization than H_2_O. However, the adsorption of H_2_O remains slightly more stable than apical O_2_, likely due to its ability to form local hydrogen bonds and a lower electrostatic repulsion, consistent with the observed adsorption energies (Table 2).

Upon the introduction of an oxygen vacancy (Figure 4c), the situation changes drastically, especially for O_2_. The inclined O_2_ configuration at the vacancy site (Figure 4d) exhibits a net charge gain of −1.28 |e|, indicating a substantial electron transfer from the ZnO surface to the O_2_ molecule. This significant charge accumulation highlights strong chemisorption and activation of the adsorbed oxygen species. This dramatic electron flow is facilitated by the local electronic enrichment of the vacancy site and the formation of strong multidentate bonds between the O_2_ and three surrounding Zn atoms. These interactions are supported by short Zn–O bond lengths (1.92–1.99 Å), and a partial reoxidation of surface Zn atoms, as evidenced by the significant charge redistribution (e.g., Zn changing from +0.66 (Figure 4c) to +1.07 (Figure 4d). Such a configuration not only enhances orbital overlap but also maximizes charge compensation, explaining the very strong adsorption energy (−2.63 eV and −2.33 eV at 12% and 25% of coverage, respectively).

In contrast, the adsorption of H_2_O near the vacancy site is destabilized, as the loss of lattice oxygen reduces available sites for hydrogen bonding. The molecule still receives some electron density, but this is insufficient to counterbalance the geometric and electronic disruption caused by the vacancy, leading to weaker adsorption.

In summary, the stability order on the ZnO (001) surface is governed by the magnitude of the charge transfer: on the pristine (001) surface, both O_2_ and H_2_O receive electrons, with H_2_O being more stabilized due to its geometry and bonding nature; on the vacancy-containing surface, O_2_ becomes a strong electron receptor, favoring its chemisorption through multizinc coordination, whereas H_2_O adsorption becomes energetically less favorable due to the disrupted local bonding environment. This trend echoes observations on ZnO [35] and other oxide surfaces such as anatase TiO_2_ (001) [36], where oxygen vacancies also act as active sites that enable exothermic O_2_ adsorption and promote the formation of superoxide or peroxide species.

#### 3.2.2. Adsorption Characteristics of H_2_O and O_2_ on ZnO (110) Surface

The pristine ZnO (110) surface was studied at two coverages 25% and 50% with the O_2_ molecule placed in its most stable inclined configuration. The adsorption energies (Table 4) reveal a weak or even unfavorable interaction for O_2_, with a slightly negative value at 25% coverage (−0.167 eV) that turns positive at 50% coverage (+0.461 eV), indicating desorption or repulsion under higher surface saturation. As the surface becomes more saturated, the O_2_–surface interaction weakens further, consistent with limited available adsorption sites.

For H_2_O, the adsorption energies on the pristine (110) surface are significantly stronger, reaching −1.585 eV at 25% coverage and −0.954 eV at 50% coverage. As the surface becomes more saturated, the adsorption weakens slightly but remains strongly exothermic, indicating that water maintains a good affinity for the ZnO (110) surface even at higher coverages.

On introducing oxygen vacancies, the surface reactivity towards O_2_ dramatically increases (Table 5). Four configurations were tested, but the most stable adsorption was found when O_2_ binds in the inclined position directly over the vacancy site, yielding strongly exothermic adsorption energies (−1.81 eV to −1.84 eV). These values represent a substantial enhancement compared to both the pristine surface and the inclined-on-Zn configuration, where adsorption remains weak (around −0.21 to −0.22 eV). As the surface becomes more saturated, O_2_ adsorption on the vacancy site remains robust, confirming the role of vacancies as key reactive centers. For H_2_O, adsorption on the vacancy surface yields moderate adsorption energies (−0.74 to −0.66 eV), which are weaker than on the pristine surface (−0.95 to −1.58 eV). This indicates that, unlike O_2_, the presence of vacancies destabilizes water adsorption.

**Figure 5:**
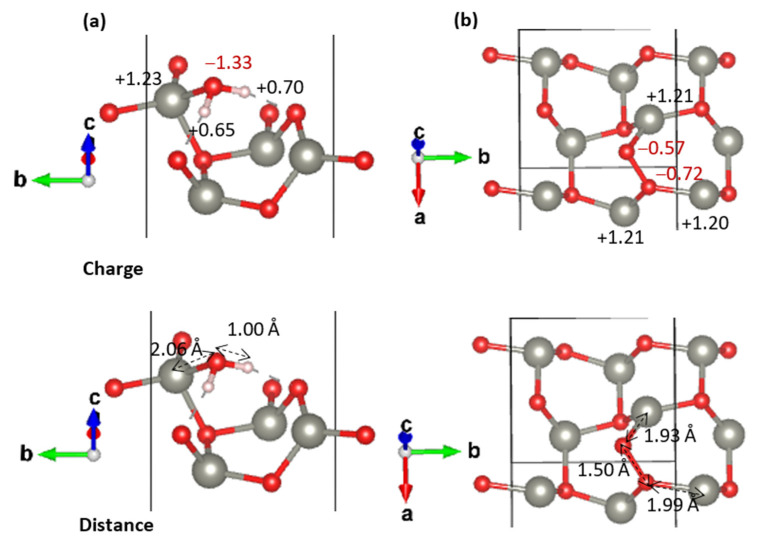
Optimized geometries and charge distributions for H_2_O and O_2_ adsorption on the ZnO (110) 1 *×* 1 surface: (**a**) H_2_O on pristine, and (**b**) O_2_ (inclined) adsorbed on the vacancy fold.

As illustrated in Figure 5a, the strong H_2_O adsorption energy on the pristine (110) surface is corroborated by the formation of two hydrogen bonds between the adsorbed water molecule and nearby surface oxygen atoms. These interactions, rather than substantial electron transfer (net charge ≈ −0.03 |e| from the surface to H_2_O), account for the adsorption stability. In contrast, O_2_ adsorption on the defective surface (Figure 5b) reveals a behavior strikingly similar to that observed on the (001) surface: the inclined_vacancy configuration adopts a three-fold coordination with surface Zn atoms and shows a comparable net charge gain of about −1.29 |e|, further stabilizing the adsorption.

In summary, the adsorption behavior on the ZnO (110) surface reinforces the general trends observed on the (001) facets. Oxygen vacancies significantly enhance O_2_ chemisorption by enabling multi-Zn coordination and facilitating strong electron transfer. Conversely, H_2_O adsorption is primarily stabilized through hydrogen bonding with surface oxygen atoms rather than charge transfer. The presence of these surface oxygen atoms on the (110) facet facilitates the formation of multiple H-bonds, making water adsorption particularly favorable—an effect visually evident on this facet but less pronounced on (001), where such bonding opportunities are limited due to the absence of surface oxygen atoms in the first atomic layer.

#### 3.2.3. Adsorption Characteristics of H_2_O and O_2_ on ZnO (100) Surface

Following the analysis performed for the (001) and (110) surfaces, we investigated the adsorption of O_2_ on the ZnO (100) surface. Given that the inclined configuration was identified as the most stable on (001) and confirmed to be energetically preferred for the 1 × 1 (100) supercell, we focused exclusively on the inclined O_2_ orientation for all coverages on this facet. Adsorption energies were computed at three surface coverages, 100%, 50%, and 25%, using 1 × 1, 2 × 1, and 2 × 2 supercells, respectively. The adsorption energy results (Table 6) reveal that O_2_ adsorption on the (100) surface is generally weak and even slightly unfavorable, with positive adsorption energies at all coverages.

These positive values indicate that O_2_ does not spontaneously adsorb on the pristine (100) surface under the tested conditions. Furthermore, the adsorption energy becomes increasingly positive as the coverage decreases, suggesting that lowering the molecular density does not enhance the interaction strength, unlike on the previous surfaces. The computed adsorption energies for H_2_O indicate a favorable interaction at high and medium coverages, but a slightly unfavorable interaction at the lowest coverage.

To assess the effect of oxygen vacancies, the same coverages were evaluated with two O_2_ configurations: inclined on Zn sites and inclined on the vacancy site. The results (Table 7) show that oxygen vacancies dramatically enhance O_2_ adsorption, especially at the vacancy fold, where adsorption energies reach −1.96 to −2.28 eV, compared to only −0.21 to −0.37 eV on regular Zn sites. For H_2_O, adsorption on the vacancy surface exhibits moderately favorable binding energies (−0.45 to −0.74 eV), slightly weaker than on the pristine surface at higher coverages. This behavior, consistent with trends observed on the (001) and (110) facets, suggests that oxygen vacancies preferentially enhance O_2_ chemisorption while having a neutral or slightly destabilizing effect on H_2_O.

**Figure 6:**
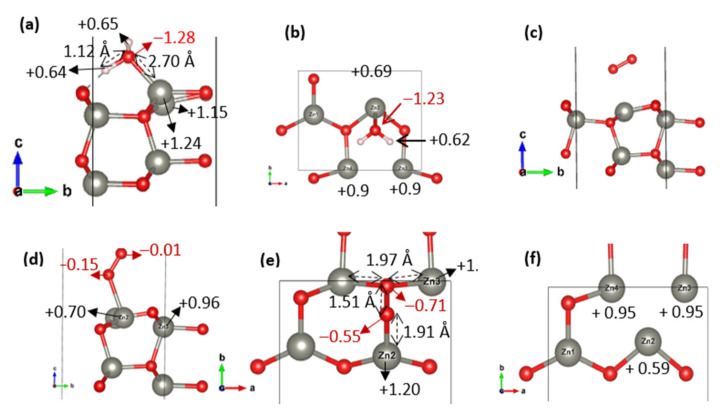
Optimized geometries and charge distributions for H_2_O and O_2_ adsorption on the ZnO (100) 2 × 1 surface: (**a**,**b**) H_2_O on pristine and vacancy sites, (**c**,**d**) O_2_ (inclined) on Zn sites of pristine and vacancy surfaces, and (**e**) O_2_ (inclined) adsorbed on the vacancy fold and (**f**) top view of vacancy.

As on the previous surfaces, this reduced stability is attributed to the loss of hydrogen bonding opportunities. Indeed, on the pristine surface (Figure 6a), H_2_O forms a hydrogen bond between one of its hydrogen atoms and a surface oxygen, which compensates for the absence of significant charge transfer. In contrast, the removal of surface oxygen atoms on the vacancy site (Figure 6b) prevents this interaction, leading to a comparable charge transfer (~0.00 |e|) but a less favorable adsorption environment.

In the case of O_2_, the pristine surface (Figure 6c) shows weak physisorption, with the molecule situated far from the surface and no significant charge exchange, consistent with the unfavorable adsorption energies in Table 7. By contrast, on the vacancy surface (Figure 6d), O_2_ adopts a closer geometry with visible interaction, receiving approximately −0.16 |e| from the surface. The most stable configuration is observed when O_2_ bridges three Zn atoms in the vacancy region (Figure 6e), where a substantial charge transfer of −1.21 |e|from the surface to the molecule occurs, enabling strong chemisorption. As observed on the (001) surface, the interaction of O_2_ with three-fold coordinated Zn atoms is supported by short Zn–O bond lengths (1.91–1.97 Å) and by a partial reoxidation of surface Zn atoms, as evidenced by the significant charge redistribution (e.g., Zn changing from +0.59 (Figure 6f) to +1.20 (Figure 6e) |e|).

In summary, the ZnO (100) surface exhibits adsorption behavior similar to that of the (001) facet: oxygen vacancies strongly enhance O_2_ chemisorption, while H_2_O adsorption remains only moderately affected. On the pristine (100) surface, H_2_O is stabilized primarily through hydrogen bonding, visibly amplified due to the presence of surface oxygen atoms in the first atomic layer, as on (110) and unlike on (001), where such an interaction is weaker and less apparent. For O_2_, while it interacts very weakly with the pristine surface, it clearly receives a significant charge from the surface when adsorbed at the vacancy fold, enabling strong chemisorption through multizinc coordination, as observed on (001), but with a reversed charge transfer direction.

Overall, H_2_O preferentially adsorbs on pristine ZnO surfaces through hydrogen bonding, with a stability trend of (110) > (001) > (100), consistent with adsorption energies of −1.58 eV, −1.18 eV, and −0.83 eV, respectively. In contrast, O_2_ adsorption is unfavorable on pristine facets but strongly enhanced at oxygen vacancies, especially on the (001) surface (−2.63 eV), followed by (100) (−2.28 eV) and (110) (−1.84 eV). This enhancement is driven by charge transfer processes facilitated by vacancy-induced active sites. These trends are in good agreement with previous findings by Zhang et al. [35], who reported H_2_O adsorption energies of −1.20 eV on both (100)/(200) and (110) surfaces, and −0.66 eV on (001), reflecting the same general stability order observed in our work. For O_2_, their results also show stronger adsorption on the (001) surface (−2.88 eV) and significantly weaker interactions on (100)/(200) (−0.31 eV) and (110) (−0.46 eV), which confirms the selective enhancement of O_2_ adsorption on the (001) facet and the relatively low reactivity of the other two facets in pristine conditions.

### 3.3. Co-Adsorption: ROS Formation

To evaluate the thermodynamic stability of O_2_ and H_2_O co-adsorption on ZnO surfaces, two adsorption energy expressions were used. The first, $E_{coadsp}^{1}$, measures the overall stabilization of the co-adsorbed system relative to separate species (Single adsorption). The second,$\;E_{coadsp}^{2}$, isolates the stabilization gained by O_2_ in the presence of pre-adsorbed H_2_O—a key descriptor for potential reactive oxygen species (ROS) formation.

The computed values (see Table 8) reveal distinct behaviors across facets. On the (001) surface (modeled at 2 × 2, 25% pristine), co-adsorption yields a significant energy gain ${(∆E}_{coadsp}^{1})$ of −1.02 eV compared to separate adsorption, indicating strong synergistic stabilization. Further decomposition shows that O_2_ contributes ${(∆E}_{coadsp}^{2})$ −0.21 eV to this gain, while the remaining −0.81 eV is attributed to H_2_O’s enhanced stability upon O_2_ co-adsorption.

For the (100) facet (2 × 1 50% pristine), the co-adsorption energy gain is more moderate, ${∆E}_{coadsp}^{1}$= −0.54 eV, with O_2_ actually destabilizing slightly (${∆E}_{coadsp}^{1}$= +0.29 eV), meaning the stabilization mainly arises from H_2_O. Conversely, on the (110) surface (1 × 2 25% pristine), the co-adsorption system is overall unstable, as reflected by a slightly positive ${∆E}_{coadsp}^{1}\;$ of +0.03 eV. This instability arises despite the fact that the individual adsorption of both O_2_ and H_2_O is energetically favorable. A detailed breakdown reveals that, within the co-adsorption configuration, O_2_ actually contributes a destabilizing effect of +1.66 eV, effectively counteracting the stabilizing influence of H_2_O and making O_2_ the primary factor responsible for the lack of overall stability in this system.

These calculations were intentionally carried out on pristine surfaces, as they provide ample adsorption space and, importantly, because H_2_O adsorption is inherently more stable on pristine facets—a prerequisite for facilitating dissociation processes required for ROS generation. Additionally, the selected supercells represent the most stable configurations for each surface when accommodating both H_2_O and O_2_, ensuring the observed trends reflect intrinsic surface-adsorbate interactions rather than geometric artifacts.

Building on the co-adsorption analysis, we now focus on the initial steps leading to reactive oxygen species (ROS) formation. Since only the (001) and (100) pristine ZnO surfaces exhibit thermodynamically stable co-adsorption of H_2_O and O_2_, the following exploration concentrates exclusively on these two facets. We specifically investigate the dissociation of the adsorbed H_2_O molecule and the subsequent proton transfer, either toward the ZnO surface or onto the co-adsorbed O_2_, leading to the generation of distinct ROS, such as hydroxyl (•OH), superoxide (•O_2_*^−^*), and perhydroxyl radical (•O_2_H). Notably, for surfaces like (001), which lack surface oxygen in their first atomic layer, only (•O_2_H) can form, as the proton transfer pathway toward surface oxygen is not available.

**Figure 7:**
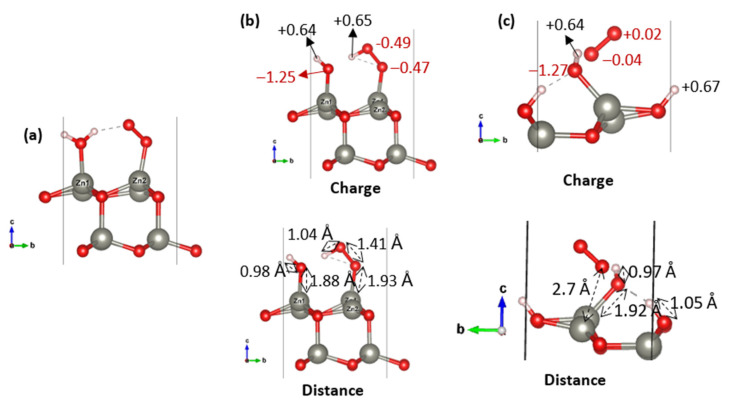
Optimized geometries illustrating (**a**) H_2_O and O_2_ co-adsorption on 2 × 2 (001), and subsequent ROS formation •O_2_H and •OH (**b**), •OH (**c**).

Energetically, the results (Table 9) show that on the ZnO (001) surface, the formation of •O_2_H and •OH radicals (Figure 7b) is favored, with a total adsorption energy of −3.07 eV, more stable than the initial co-adsorption configuration (−2.87 eV, Figure 7a). This suggests a spontaneous and energetically favorable transformation of adsorbed H_2_O and O_2_ into reactive oxygen species. The Bader charge analysis (Figure 7b) further supports this, with the oxygen atoms in •O_2_H carrying partial negative charges of −0.49 and −0.47 |e|, and the protonated oxygen reaching −1.25 |e|, consistent with the formation of hydroperoxyl and hydroxyl radicals.

In contrast, on the ZnO (100) surface, only the •OH formation appears to occur. The final configuration (Figure 7c) shows a slight stabilization (−1.09 eV) compared to co-adsorption (−1.00 eV). However, the Bader charge analysis reveals a negligible charge transfer to the O_2_ molecule (−0.04 and +0.02 |e| on the two oxygen atoms), leading to a total of −0.02 |e|. Combined with the relatively long O_2_–Zn distance of 2.70 Å, this indicates that O_2_ remains weakly physiosorbed and does not form a superoxide •O_2_^−^ species. Only the •OH radical is realistically formed on this surface.

When a surface oxygen vacancy is introduced on the (001) facet, the formation of •O_2_H and •OH remains exothermic (−2.64 eV) but becomes less favorable compared to the pristine case (−3.07 eV). This suggests that while the vacancy facilitates stronger O_2_ adsorption, it does not enhance the co-adsorption-driven ROS generation.

As the •O_2_H formation is energetically unfavorable (+1.99 eV), only the •OH species is suggested to be realistically accessible. Overall, these trends highlight that while both surfaces support ROS formation, their distinct atomic arrangements direct the system toward specific ROS types.

## 4. Conclusions

This work provided a comprehensive investigation of H_2_O and O_2_ adsorption on the three main ZnO surfaces—(001), (100), and (110)—in both pristine and oxygen-defective states, with a particular focus on co-adsorption and reactive oxygen species (ROS) formation. In the present computational framework, the focus is placed on capturing the fundamental processes governing surface adsorption and reactivity under well-defined conditions. Although light irradiation, a central factor in photocatalysis, is not explicitly considered here, the adopted approach provides a robust basis for disentangling the intrinsic adsorption mechanisms.

We first showed that H_2_O adsorption was more stable on pristine surfaces, with the (110) facet showing the highest affinity (−1.58 eV), followed by (001) and (100). This trend correlates with surface morphology: (110) exposed surface oxygen atoms that favored hydrogen bonding, unlike (001), which lacked them. In contrast, O_2_ adsorption was weak on pristine surfaces but became significantly enhanced in the presence of oxygen vacancies, particularly on the (001) facet (−2.63 eV), due to a defect-induced charge transfer.

Next, the co-adsorption of H_2_O and O_2_ was analyzed. Only the (001) and (100) pristine surfaces showed stable co-adsorption, with energy gains of −1.02 eV and −0.54 eV, respectively. On (001), both molecules contributed to stabilization, while on (100), the gain was mostly due to H_2_O. The (110) surface, however, displayed a slightly unstable co-adsorption (+0.03 eV), mainly because of O_2_’s destabilizing effect.

Focusing on ROS formation, we found that the (001) surface enabled the spontaneous generation of •OH and •O_2_H radicals, with a final energy of −3.07 eV, more favorable than the initial co-adsorbed state. In contrast, the (100) surface only formed •OH, and O_2_ remained weakly physiosorbed due to a minimal charge transfer. Notably, introducing an oxygen vacancy on (001) strengthened O_2_ adsorption but slightly reduced ROS formation energy (−2.64 eV), indicating that defects enhance O_2_ activation but not co-adsorption-driven reactivity.

In summary, this study highlighted the crucial interplay between surface structure, defect presence, and molecule–molecule interactions in governing ZnO surface reactivity. The (001) pristine surface emerged as the most promising candidate for spontaneous and efficient ROS formation, particularly of •OH and •O_2_H radicals, owing to its favorable co-adsorption behavior and H_2_O dissociation capacity. These findings provide essential insights for the rational design of reactive ZnO surfaces for applications such as photocatalysis, pollutant degradation, and gas sensing.

## Figures and Tables

**Table 1:** Surface area (*A*), surface energy (*γ*), and surface thickness (*L*) of studied ZnO facets.

Surface	$A({Å}^{2})$	$\gamma (J/m^{2})$	*L* (Å)
(010)	16.82	1.01	12.16
(100)	16.82	1.01	12.16
(001)	9.10	1.83	13.58
(110)	29.13	1.08	9.72
(102)	24.78	1.66	10.61
(111)	30.52	1.65	10.23
(112)	34.35	2.89	10.67
(101)	19.12	2.69	11.23

**Table 2:** Adsorption energies of O_2_ and H_2_O on pristine ZnO (001) at various coverages.

Coverage_Pristine	$E_{O2}^{Apical}$ (eV)	$E_{O2}^{inclined}$ (eV)	$E_{H2O}$ (eV)
2 × 4_12%	−0.59	−1.11	−0.83
2 × 2_25%	−0.57	−1.04	−0.81
1 × 2_50%	−0.51	−0.81	−0.87

**Table 3:** Adsorption energies of O_2_ and H_2_O on ZnO (001) surface with oxygen vacancies ($E_{O2}^{Apical\_Zn}$, $E_{O2}^{inclined\_Zn}$, $E_{O2}^{Apical\_Vac}$, $E_{O2}^{inclined\_Vac}$, and $E_{H2O}$ corresponding respectively to apical and inclined configurations on Zn sites and vacancy sites, and H_2_O adsorption near vacancy).

Coverage Vacancy	$E_{O2}^{Apical\_Zn}$ (eV)	$E_{O2}^{inclined\_Zn}$ (eV)	$E_{O2}^{Apical\_Vac}$ (eV)	$E_{O2}^{inclined\_Vac}$ (eV)	$E_{H2O}$ (eV)
2 × 4_12%	−0.83	−0.90	−1.50	−2.63	−0.49
2 × 2_25%	−0.41	−0.96	−0.89	−2.33	−0.56
1 × 2_50%	−0.26	−0.75	−1.05	−1.98	−0.66

**Table 4:** Adsorption energies of O_2_ and H_2_O on pristine ZnO (110) at various coverages.

Coverage	$E_{O2}^{inclined}$ (eV)	$E_{H2O}$ (eV)
1 × 2_25%	−0.167	−1.585
1 × 1_50%	+0.461	−0.954

**Table 5:** Adsorption energies of O_2_ and H_2_O on ZnO (110) surface with oxygen vacancies.

Coverage	$E_{O2}^{inclined\_Zn}$ (eV)	$E_{O2}^{inclined\_Vac}$ (eV)	$E_{H2O}$ (eV)
1 × 2_25%	−0.22	−1.81	−0.74
1 × 1_50%	−0.21	−1.84	−0.66

**Table 6:** Adsorption energies of O_2_ and H_2_O on pristine ZnO (100) at various coverages.

Coverage	$E_{O2}^{inclined}$ (eV)	$E_{H2O}$ (eV)
2 × 2_25%	+1.60	+1.24
2 × 1_50%	+0.37	−0.83
1 × 1_100%	+0.26	−1.18

**Table 7:** Adsorption energies of O_2_ and H_2_O on ZnO (100) surface with oxygen vacancies.

Coverage_Vacancy	$E_{O2}^{inclined\_Zn}$ (eV)	$E_{O2}^{inclined\_Vac}$ (eV)	$E_{H2O}$ (eV)
2 × 2_25%	−0.21	−1.96	−0.74
2 × 1_50%	−0.37	−2.28	−0.73
1 × 1_100%	−0.23	−2.04	−0.45

**Table 8:** Co-adsorption energies of H_2_O and O_2_ on pristine ZnO surfaces (001, 100, 110): $E_{coads}^{1}$ (total co-adsorption energy), $E_{H2O+O2}$ (sum of separate adsorption), ${∆E}_{coads}^{1}$ (co-adsorption gain/loss), $E_{coads}^{2}$ (O_2_ stabilization with pre-adsorbed H_2_O), ${∆E}_{coads}^{2}$ (O_2_’s energetic contribution).

Facets	$E_{coads}^{1}$	$E_{H2O+O2}$	${∆E}_{coads}^{1}$	$E_{coads}^{2}$	${∆E}_{coads}^{2}$
(001)	−2.87	−1.85	−1.02	−2.06	−0.21
(100)	−1.00	−0.46	−0.54	−0.17	+0.29
(110)	−1.72	−1.75	+0.03	−0.13	+1.66

**Table 9:** Energetic assessment of ROS formation on (001) and (100) pristine ZnO surfaces: $E_{OH\_}^{ROS}$ and $E_{OH\_OOH}^{ROS}$ represent the formation energies of •OH and •OH + •O2H ROS species, respectively.

Facets	$E_{coadsp}^{1}$	$E_{OH}^{ROS}$	$E_{OH\_O2H}^{ROS}$
(001)	−2.87	_	−3.07
(100)	−1.00	−1.09	+1.99

## Data Availability

The original contributions presented in this study are included in the article/Supplementary Material. Further inquiries can be directed to the corresponding author.

## Supplementary Materials

The following supporting information can be downloaded at https://www.mdpi.com/article/10.3390/nano15171328/s1, Figure S1. Slab cuts for surfaces (001), (100), (101) and (111) from the bulk ZnO. Figure S2. Supercell Models of Pristine ZnO.
