# Supplementary material for: Computational Study of ZnO Surface Catalysis: Adsorption of H2O or/and O2 as a Pathway to ROS Formation
Source: Nanomaterials (Basel). 2025 Aug 29;15(17):1328. doi: 10.3390/nano15171328 (PMC12429867; doi:10.3390/nano15171328)
Supplement: Supplementary file 1 [file nanomaterials-15-01328-s001.zip › nanomaterials-3816344-supplementary.pdf]

# Computational Study of ZnO Surface Catalysis: Adsorption of $H_2O$ or/and $O_2$ as a Pathway to ROS Formation.

**S. E. Adjovi [1], M. Calatayud[1], L. Gracia[2]**

[1] Sorbonne Université, MONARIS, CNRS-UMR 8233, 4 Place Jussieu, F-75005 Paris.

[2]Departament de Química Física, Universitat de València, 46010 Burjassot, Spain

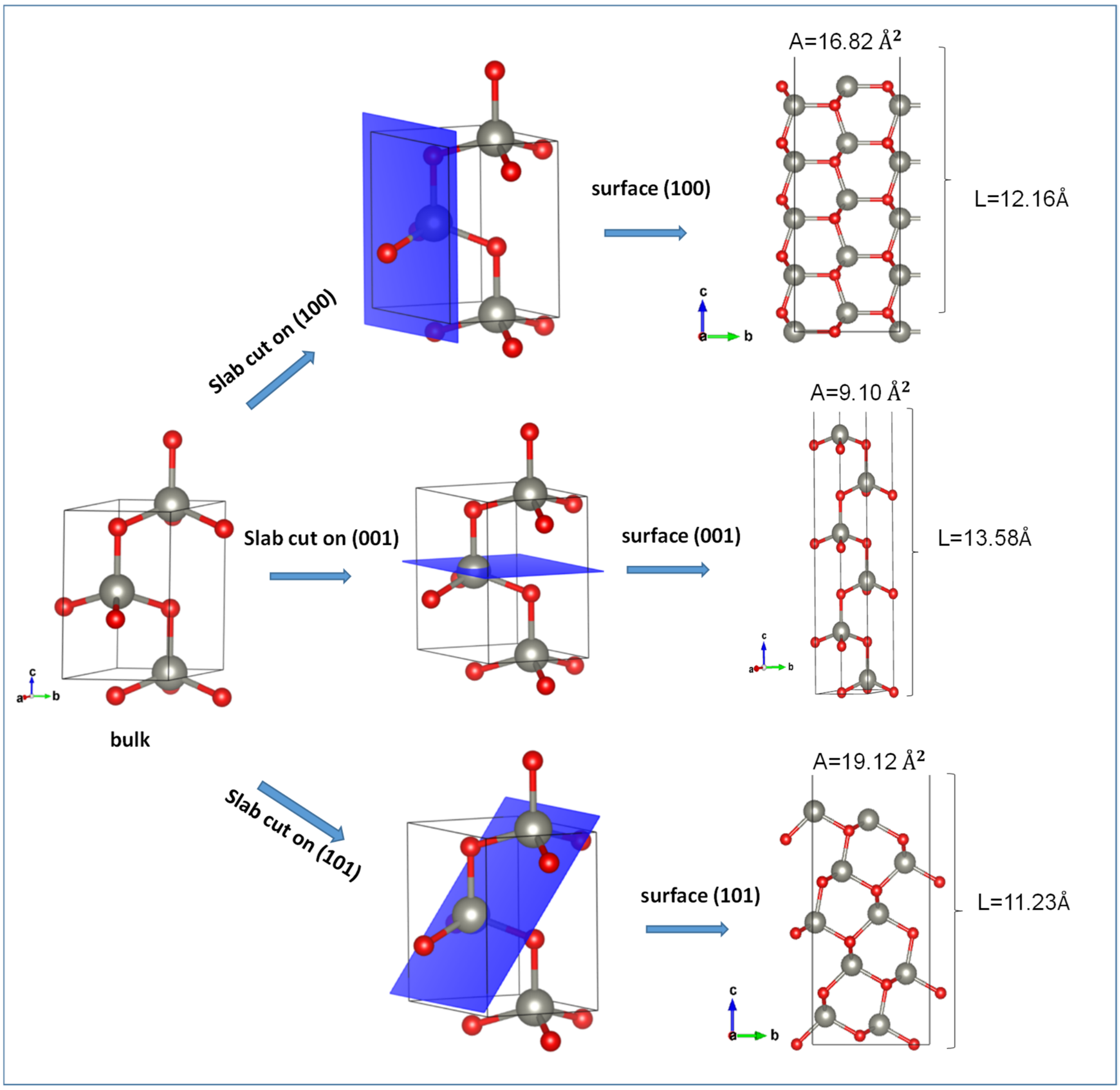


**Figure S1.** Slab cuts for surfaces (001), (100), (101) and (111) from the bulk ZnO.

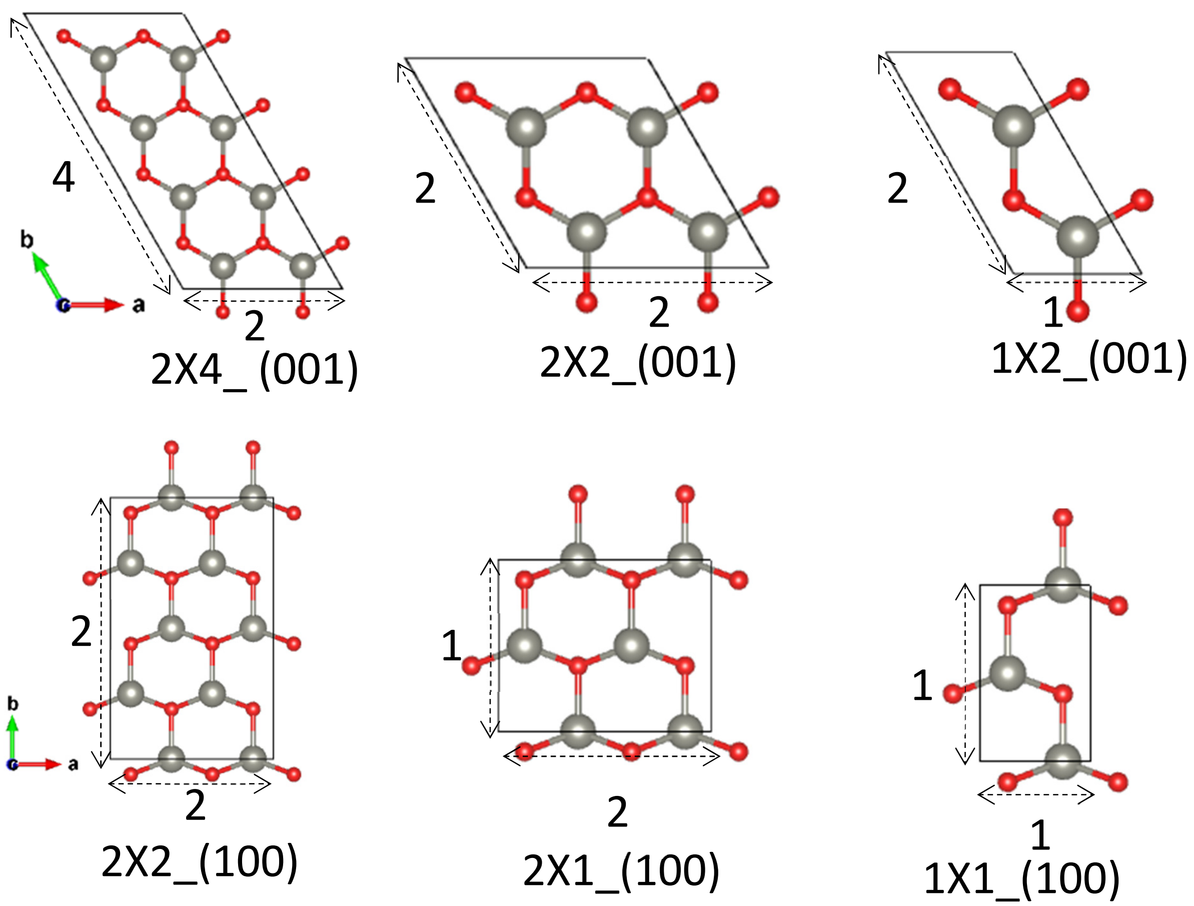


**Figure S2.** Supercell Models of Pristine ZnO
